# Mapping Soil Surface Macropores Using Infrared Thermography: An Exploratory Laboratory Study

**DOI:** 10.1155/2014/845460

**Published:** 2014-10-14

**Authors:** João L. M. P. de Lima, João R. C. B. Abrantes, Valdemir P. Silva, M. Isabel P. de Lima, Abelardo A. A. Montenegro

**Affiliations:** ^1^Department of Civil Engineering, Faculty of Science and Technology of the University of Coimbra (FCTUC), Rua Luís Reis Santos, Campus II, University of Coimbra, 3030-788 Coimbra, Portugal; ^2^Institute of Marine Research, IMAR/MARE, Coimbra, Portugal; ^3^Department of Agricultural Engineering, Rural Federal University of Pernambuco, 52171-900 Recife, PE, Brazil

## Abstract

Macropores and water flow in soils and substrates are complex and are related to topics like preferential flow, nonequilibrium flow, and dual-continuum. Hence, the quantification of the number of macropores and the determination of their geometry are expected to provide a better understanding on the effects of pores on the soil's physical and hydraulic properties. This exploratory study aimed at evaluating the potential of using infrared thermography for mapping macroporosity at the soil surface and estimating the number and size of such macropores. The presented technique was applied to a small scale study (laboratory soil flume).

## 1. Introduction

Macropores and water flow in soils and substrates are complex, are related to topics like preferential flow, nonequilibrium flow, and dual-continuum, and have been addressed by many studies in the last decades (e.g., the reviews by Beven and Germann in 1982 [1] and, most recently, 30 years later, in 2013 [2]). Since macropores affect soil permeability, they directly influence other hydrological processes (e.g., surface runoff and associated transport processes).

The water movement and the fertilizers, pesticides, and other pollutants transporting in the soil through macropores have significant impact on hydrological response and water quality (e.g., [3]). These structures convey water to greater depths with higher speed, thus influencing water infiltration into the soil and solute transport. The macropores also directly affect the air flow into the soil, the plants root growth, and biological activity (e.g., [4–6]). Therefore, a high macroporosity enhances air and water movement in the soil, promoting also infiltration and root penetration.

Recently, infrared thermography has been successfully applied as a tool for high resolution imaging in different hydrological studies, conducted at quite different spatial scales. Thermographic techniques are based on records of bodies' temperatures, which are taken at certain instants or over time and at a given space scale; this technology is allowing for a nonconventional acquisition of data and analysis of different processes and their time and space dynamics.

The use of portable infrared cameras or thermal imaging cameras has gained popularity due to their easy handling and adjustment of the vision field to a specific study area. Mejías et al. [7] and Danielescu et al. [8] used infrared cameras mounted in aircrafts for mapping groundwater discharge in shallow estuaries, provided that there is a thermal contrast between groundwater and the receiving surface waters. Cardenas et al. [9] characterized the thermal heterogeneity in a small stream during different flow conditions. Pfister et al. [10] used ground-based thermal imagery as a simple, practical tool, for mapping saturated area connectivity and dynamics. It was possible to discriminate between areas with snow cover, snow melt, soil seepage, and stream water. It was possible to detect when and where variably saturated areas were active and when connectivity existed between the hillslope-riparian-stream systems. This was a simple and inexpensive technology for sequential mapping and characterisation of surface saturated areas and a useful complement to conventional tracer techniques. Schuetz et al. [11] used infrared thermal imaging combined with the injection of heated water as an artificial tracer technique to characterize the spatial distribution of flow paths and to assess transport properties in a 65 m^2^ experimentally constructed wetland with water depths between 0.1 and 0.2 m. For the studied conditions, the authors observed that heated water can be used as a conservative artificial tracer for plot scale experiments.

Infrared thermographic techniques studied in laboratory conditions were used to assess different surface hydrological processes. A technique to estimate soil surface microrelief and rill morphology using infrared thermography is presented in de Lima and Abrantes [12]. The authors were able to generate 3D models of soil surface elevation for both bare soil and soil covered with organic residue. In de Lima and Abrantes [13] the authors estimated very shallow flow velocities (overland and rill flow), by injecting a thermal tracer (e.g., heated water) into very shallow flows and visualizing the leading edge of the tracer by means of infrared video. Soil flume laboratory experiments have been conducted for many years aiming at studying specific processes and interactions in controlled conditions allowing for repetitions in a short period of time (e.g., [14–19]).

*In situ* small scale infiltration tests (e.g., profile or plot scale), under saturated or unsaturated soil conditions, normally have difficulty to cope with macropores. The quantification of the number of macropores, which are drivers of water, and the determination of their geometry will provide a better understanding of the effects of pores on the physical and hydraulic properties of the soil (e.g., [20]). Thus, this exploratory study aimed at evaluating the potential of using infrared thermography for mapping macroporosity at the soil surface and estimating the number and size of such macropores. The presented technique was applied to a small laboratory scale study (i.e., soil flume). Thus, upscaling results to field, hillslope, and even plot scales will require further investigation, which is beyond the scope of the presented analysis.

## 2. Material and Methods

### 2.1. Laboratory Setup

**Figure 1:**
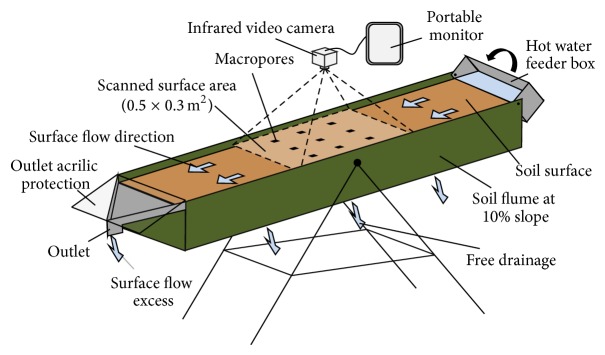
Sketch of the laboratory setup using a soil flume and an infrared video camera.

A schematic representation of the used exploratory laboratory setup is shown in Figure 1. The experiments were carried out using a 3.00 m long, 0.30 m wide, and 0.12 m deep free drainage soil flume, set at a 10% slope (round number also used in many other works). A feeder box installed at the upslope end of the flume allowed the application of a defined volume of hot water uniformly over the soil surface. A rectangular measuring area (0.50 × 0.30 m^2^) with macropores was defined at the flume soil surface, approximately 0.5 m downstream of the hot water feeder box.

The experiments were carried out using a loamy-sand soil collected from the River Mondego banks (Coimbra, Portugal). The soil presented 6% clay, 11% silt, 82% sand, and a 1750 kg/m^3^ bulk density. Saturated hydraulic conductivity was ~4.51 × 10^−6 ^m/s, for the macropores-free soil.

Thermal videos of the soil surface were recorded with an Optris PI-160 portable infrared video camera (Optris GmbH, Germany) with an optical resolution of 160 × 120 pixels, a thermal resolution of 0.1°C, an accuracy of ±2%, a frame rate of 100 Hz, and a lens with a view field of 23° × 17° and focal length of 10 mm. The camera was attached to a metal support structure 0.75 m above the flume soil surface with the focal length direction perpendicular to the soil surface (Figure 1).

### 2.2. Soil Surface Macropores

**Figure 2:**
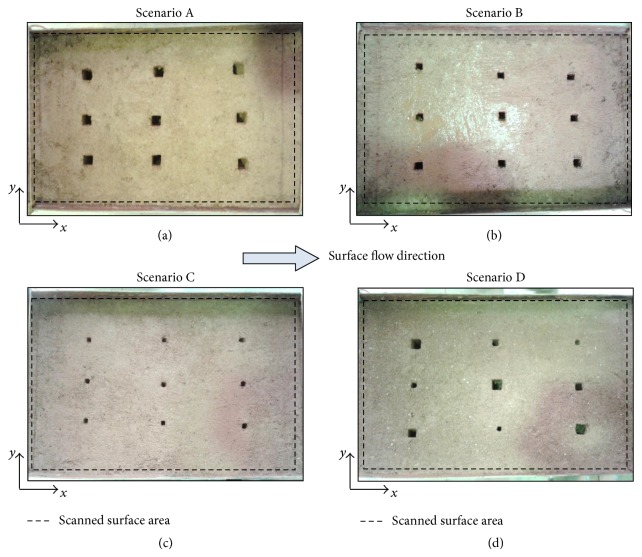
Top view (photographs) of the flume soil surface showing macropores of different sizes scattered in accordance with the four scenarios described in the text: (a) Scenario A; (b) Scenario B; (c) Scenario C; and (d) Scenario D. The *x*-axis represents the downslope distance along the length of the flume and the *y*-axis represents the distance across the width of the flume (the dashed line defines the measuring area with 0.50 × 0.30 m^2^). See Figure 1.

Soil surface vertical macropores with three different rectangular cross section areas were artificially created to test the proposed thermographic technique: (i) large macropores with an area of 256 mm^2^ (16 × 16 mm^2^); (ii) medium macropores with an area of 120 mm^2^ (12 × 10 mm^2^); and (iii) small macropores with an area of 36 mm^2^ (6 × 6 mm^2^). The tests were conducted for four different macropores spatial arrangement's scenarios; these scenarios are shown in Figure 2: (a) 9 large macropores (Scenario A); (b) 9 medium macropores (Scenario B); (c) 9 small macropores (Scenario C); and (d) a combination of 3 large, 3 medium, and 3 small macropores (Scenario D). It should be noted that field conditions are quite different from a uniform plane surface, clean of stones, debris, and vegetation, with homogeneous colours and macropores with well-defined shapes, used in this exploratory study.

### 2.3. Experimental Procedure

Air dried presieved soil was manually spread over the flume and gently tapped to obtain a soil layer with a uniform thickness of 0.10 m with a bulk density of ~1750 kg/m^3^. A sharp straight-edged blade was used to produce a smooth plane soil surface. The soil was saturated and left to dry, aiming to obtain a consistency that allowed the artificial creation of vertical macropores at the soil surface; this was carried out by perforating the soil layer, throughout its 0.10 m thickness, with rectangular cross-section metal rods that were described in Section 2.2. After the creation of the macropores, the soil was again saturated.

The technique starts by applying approximately 1.5 L of heated water over the soil surface, at a temperature around 80–85°C. The water was manually released using the feeder box located upslope of the flume (Figure 1). It was applied with the lowest possible discharge in order to guarantee flow depth uniformity over the measuring area, minimum soil surface disturbance, and the unaltered structure of the macropores by the flowing water. The volume of hot water used (and applied discharge) should be adjusted to the test's conditions (e.g., dimension of the measuring area, slope, water temperature, soil permeability, and dimension of macropores).

The hot water created a wave that covered uniformly the soil surface; along the flume, part of the water flows to the macropores, part infiltrates into the soil, and part flows out of the flume through the downslope outlet, as overland flow. The hot water briefly accumulates inside the macropores before exiting freely the soil layer due to the flume free drainage (Figure 1). Since macropores were filled with flowing hot water, they present higher temperatures in the thermal videos, recorded with the infrared video camera, which allowed the mapping of their spatial location and the estimation of their approximate area. Because the soil was close to the field capacity at the beginning of the experiments, the percentage of water infiltrated into the soil was very low compared to the percentage flowing into the macropores.

Soil surface thermal videos were recorded with the infrared camera throughout the experiments. However, thermograms of the surface of the overland flow layer did not provide any spatial variability of temperature. Therefore, for each scenario (Figure 2), a thermogram of the soil surface was selected, corresponding to an instant just after (approximately 30 s) the passage of the wave of hot water through the scanned area; thermogram is a graphic record of temperature variations, and it represents radiation in the infrared range of the electromagnetic spectrum, providing identification of pixels associated with different surface temperatures. Because, in general, macropores present higher temperatures, a threshold temperature (*τ*) can be selected, which allows identifying the pixels associated with the macropores (i.e., pixels with temperature values above a given threshold temperature), thus distinguishing them from the remaining pixels that cover the soil surface scanned area (i.e., pixels with temperature values below the temperature threshold). The percentage of pixels that have temperature above the threshold temperature was called threshold percentage of pixels (*α*).

The number of macropores detected with this technique, as well as their position and area, depends upon the selected threshold temperature (*τ*). Higher threshold temperatures will lead to a detection of a lower number of macropores with smaller cross-section area (and, correspondingly, lower temperatures identified by the thermal images). On the contrary, lower threshold temperatures will lead to a detection of a higher number of macropores with bigger cross-section area. The lower-limit temperature from which all macropores are detected was called critical threshold temperature (*τ*_*c*_). Selected threshold temperatures below *τ*_*c*_ will only lead to an increase of the macropores' area, since the maximum number and position of macropores were already detected since the location of every pixel is known.

## 3. Results and Discussion

**Figure 3:**
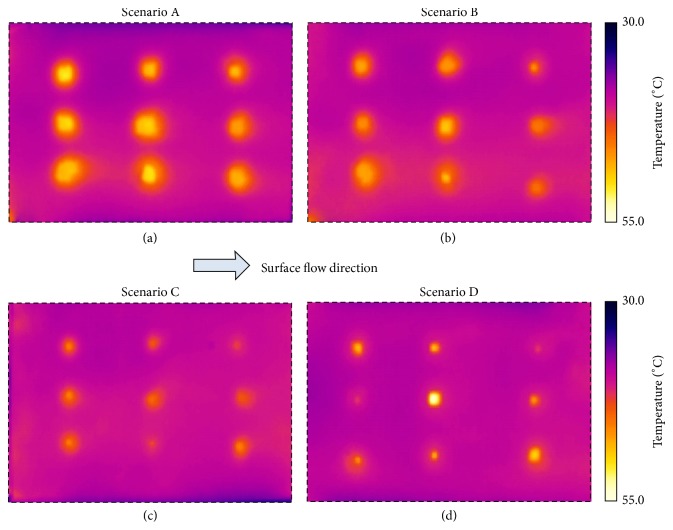
Thermograms of the soil surface for the four scenarios: (a) Scenario A; (b) Scenario B; (c) Scenario C; and (d) Scenario D. The dashed line defines the measuring area with 0.50 × 0.30 m^2^. See also Figure 2.

Thermograms for the four scenarios defined in Section 2.2 are presented in Figure 3. In the thermograms, it is possible to identify the location of the different macropores, perceptible by the presence of groups of pixels exhibiting a brighter colour. These thermal marks are the result of the higher temperatures produced by the accumulation of flowing hot water in the macropores. Despite the rectangular shape of the macropores, the thermal marks identified visually in the thermograms are of approximate circular shapes. Also, the thermal marks are, in general, larger than the actual area of the macropores. This temperature smearing around the macropores is caused by thermal diffusion, by the higher infiltration of hot water around the macropores, and by the relatively low resolution of the camera.

In general, larger macropores led to larger thermal marks, exhibiting higher temperatures at the centre caused by the accumulation of hot water. Thermograms of Scenarios A, B, and C, of Figures 3(a) to 3(c), clearly show thermal marks with different areas, which are in accordance with the different areas of the macropores of each scenario. In the thermogram of Scenario D, in Figure 3(d), it is also possible to distinguish the macropores of different areas in the same thermogram. Macropores concentrate water which flows into these hollows and are, therefore, in contact with more hot water than the surrounding soil. This is why macropores have a different temperature than the soil surface.

**Figure 4:**
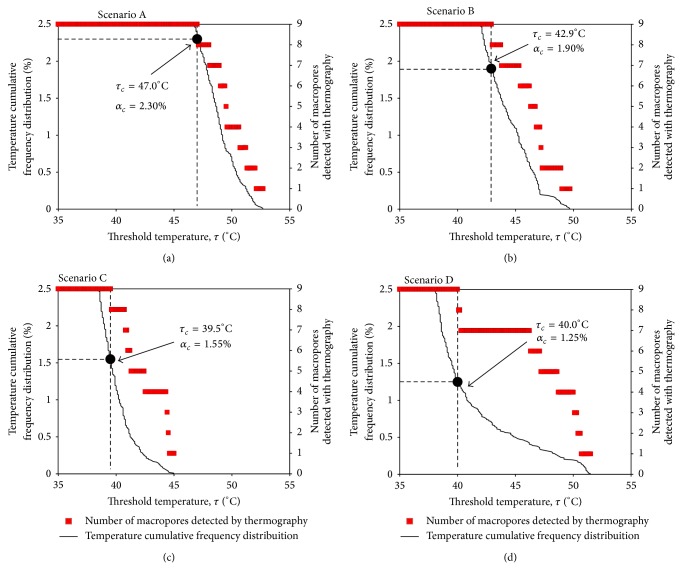
Relation between threshold temperature (*τ*) and the number of macropores detected with thermography, for the four scenarios: (a) Scenario A; (b) Scenario B; (c) Scenario C; and (d) Scenario D. Temperature cumulative frequency distribution curves are also plotted, up to 2.5%. Critical threshold temperature (*τ*_*c*_) and corresponding critical threshold percentage of pixels (*α*_*c*_) are identified.

The relation between the threshold temperature (*τ*) and the number of macropores detected using thermography is shown in Figure 4 (right axis). The same graph shows the temperature cumulative frequency distribution of the pixels' temperature (T), corresponding to the four thermograms in Figure 3, up to 2.5% (left axis). For each scenario, the critical threshold temperature (*τ*_*c*_) and corresponding critical threshold percentage of pixels (*α*_*c*_) are identified in the figure. We highlight that both *τ*_*c*_ and *α*_*c*_ depend on the area of the thermal marks in the thermograms; For example, Scenario A, with only large macropores, leads to the highest *τ*_*c*_ and *α*_*c*_. The threshold *τ*_*c*_ depends also on the temperature of the hot water applied, the initial surface temperature, and the macropores present at the soil surface. Therefore, it is not possible to specify single *τ*_*c*_ or *α*_*c*_ values, representative of all scenarios. Nevertheless, such values are essential for processing the data and mapping existing soil surface macropores. Therefore, bearing in mind the results obtained in our experiment for all the tests (Figure 4), we have further adopted a threshold percentage of pixels (*α*) of 2.5% for all scenarios, since in our tests we found always *α*_*c*_ < 2.5%. For each scenario, the corresponding temperature frequency distribution curve (Figure 4) yields a specific value of the threshold temperature (*τ*) for *α* = 2.5%, which we have used to study the area and location of the macropores, using thermography.

**Figure 5:**
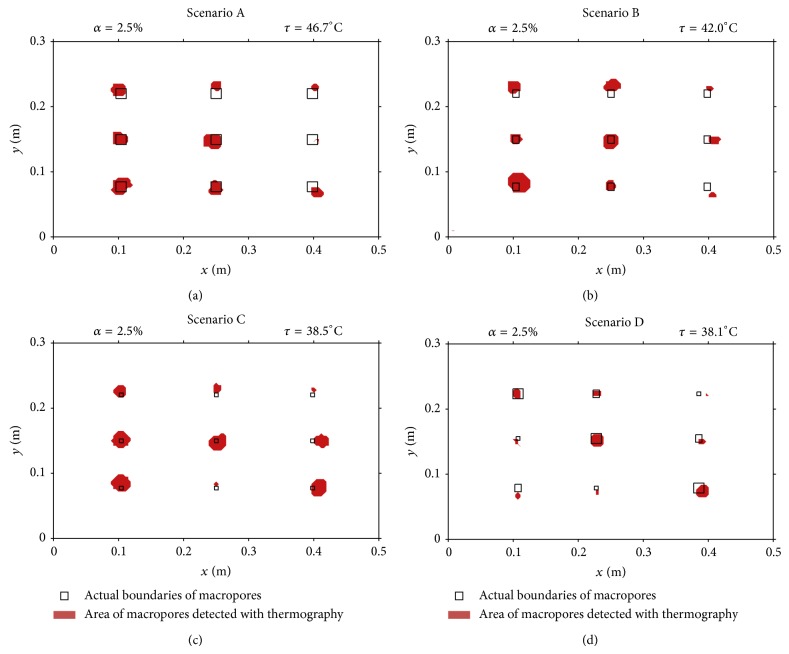
Comparison between the boundaries of the actual macropores and the area detected with thermography, for the four scenarios. Across the area scanned by the thermographic camera, the macropores are located using (*x*, *y*) coordinates. The *x*-axis represents the downslope distance along the length of the flume (0.5 m) and the *y*-axis represents the distance across the width of the flume (0.3 m). See also Figures 2 and 3.

Soil surface macropores were identified by applying to the temperature data the threshold temperature (*τ*) corresponding to *α* = 2.5%; the selected *τ* were subtracted to all temperature values from the thermograms; positive values, which correspond to pixels with temperature values above *τ*, are associated with the macropores. The comparison between the boundaries of the actual macropores and the macropores' area detected with thermography is shown in Figure 5 (top view-2D). The macropores' area detected with thermography was, in general, larger than their actual area. Therefore, the technique did not accurately estimate the actual area of the macropores, especially for the smaller ones. It is possible to observe in Figure 5(d) that the proposed technique can be used to distinguish macropores with different sizes (i.e., cross-section area), since larger macropores were detected with thermography as having also larger areas. This has already been suggested by visual observation of the thermal marks in the thermograms in Figure 3.

**Figure 6:**
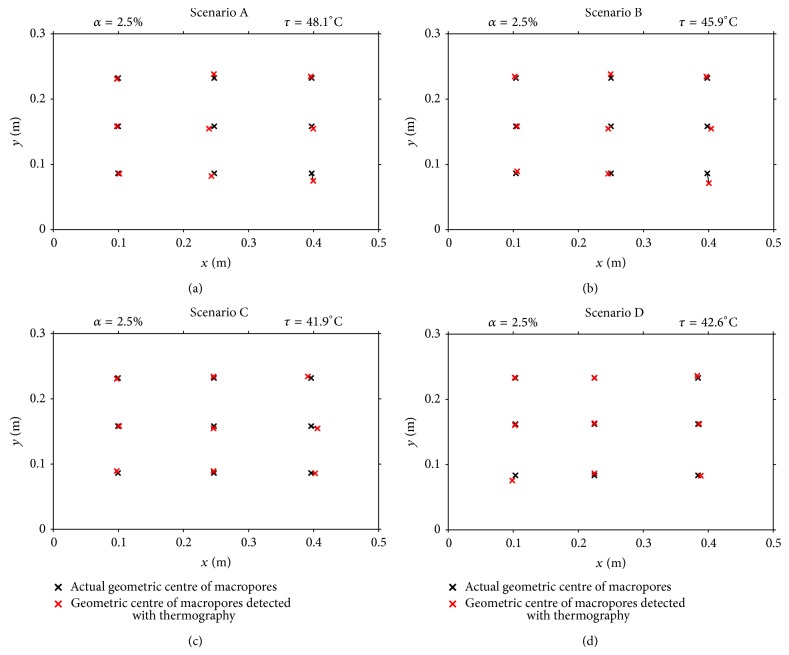
Comparison between the actual geometric centre of the macropores and their geometric centre detected using thermography, for the four scenarios. See Figure 5.

Figure 6 shows the comparison between the position of the actual geometric centre of the macropores and their geometric centre detected using thermography. In general, the proposed technique allowed the correct estimation of the macropores' location. Although the technique did not assess accurately the actual area of macropores, it clearly allowed mapping the macropores and identifying their spatial distribution and position across the studied soil surface area.

## 4. Conclusion

A novel technique to detect and characterize soil surface macropores based on infrared thermography was presented and discussed, based on exploratory laboratory soil flume experiments. For our laboratory conditions, the thermographic technique was successful in identifying the presence of macropores at the soil surface, providing a low cost and fast methodology to map soil surface macropores. Although uncertainties arise during the analysis about the macropores boundaries at the soil surface, which therefore restricts the quantification of their shape and size, the technique seems promising and able to identify macropores' spatial distribution with satisfactory accuracy.

Only extensive field work can, in fact, reveal the relevance of the technique in real (field) conditions. Future work has to be carried out to verify the applicability of the proposed technique under different field conditions, such as soil type, dimensions of scanned area, surface microrelief and roughness, presence of vegetation, stones, and other obstructions, which is beyond the scope of the presented analysis.
